# Biophosphorescence in fluorescent millipedes (Diplopoda: Xystodesmidae) and its relationships with bioluminescence

**DOI:** 10.1038/s41598-023-47860-9

**Published:** 2023-12-13

**Authors:** Vadim R. Viviani

**Affiliations:** Department of Physics, Chemistry and Mathematics, Center for Sustainable Sciences and Technologies (CCTS), UFSCar, Sorocaba, SP Brazil

**Keywords:** Biological fluorescence, Optical spectroscopy, Photobiology

## Abstract

Three types of luminescence have been reported in living organisms: bioluminescence (BL), ultraweak chemiluminescence and biofluorescence (FL). In millipedes, both BL and FL have been reported in *Motyxia sequoiae* and related Xystodesmidae species. Noteworthy, when walking at night with a UV lantern at the Biological Station of Highlands, I found three blue-fluorescent millipedes (*Deltotaria brimleii*, *Deltotoria* sp and *Euryus orestes*) that also displayed phosphorescence after turning off the UV source. The phosphorescence of the cuticle was in the green region (λ_max_ = 525 nm). The phosphorescence remained associated with cuticle and pellets, but frozen fluorescent supernatants, also displayed phosphorescence. The fluorescent compounds extracted from the cuticles in water and methanol and separated by TLC, displayed fluorescence spectra similar to that of 6-pteridine carboxylic acid. In contrast to *Motyxia sequoiae* cuticle extracts, no bioluminescence was found in *Deltatoria* and Euryus extracts  in the presence of MgATP, but weak green chemiluminescence was detected with H_2_O_2_ and superoxide. The spectral overlapping of phosphorescence of these millipedes with the bioluminescence of *Motyxia* (~ 507 nm) and the intimate association of both types of luminescence with the cuticles, raises the possibility that bioluminescence in *Motyxia* may arise from chemiluminescence reactions preferentially generating triplet excited states instead of singlet states.

## Introduction

In living organisms, three types of luminescence have been reported: (1) bioluminescence, a special case of enzymatically catalyzed chemiluminescence for communicative purposes^1^, (2) ultraweak chemiluminescence resulting from side-reactions of the oxidative metabolism^2^ and (3) fluorescence, also referred as biofluorescence, a kind of photoluminescence which depends on excitation by light. Biofluorescence, has been reported for several classes of organisms, including mainly marine organisms with fluorescent proteins (FPs), arthropods, amphibians, reptiles, birds and even mammals^3–6^. In marine organisms such as jellyfishes, GFPs participate in modulation of bioluminescence spectrum through energy transfer (BRET)^7^, and possibly as fluorescent light dissipators of damaging UV light in some corals. In some cases, such in the case of bird´s plumage, in which fluorescence enhances the plumage color effect for sexual attraction purposes^8,9^, the biological function of fluorescence is evident. In some insects and amphibians, fluorescence may also display an amplification effect on visual clues. In other cases, such as the scorpion´s cuticle, fluorescence does not have any apparent function, being just a side effect of production of cuticle fluorescent compounds^10^.

Bioluminescence occurs in several organisms, both marine and terrestrial. It arises from exothermic oxidative reactions in which compounds generally called luciferins are oxidized by molecular oxygen in the presence of enzymes called luciferases or photoproteins, producing peroxidic intermediates, whose spontaneous cleavage preferentially yield singlet (fluorescent state) excited products that decay, emitting light^1^. Among arthropods, it occurs in marine crustaceans, millipedes and mainly insects^11^.

Despite some confusion in the old literature regarding the use of the term phosphorescence for bioluminescence, until recently phosphorescence was not known to occur among living organisms or biological materials. Recently, phosphorescence has been reported in rice, starch and frozen serum albumin solutions^12^. An image of phosphorescence has been published in millipedes of Xystodermidae family, but no detailed characterization of this phosphorescence has been carried out yet^13^.

In millipedes, bioluminescence was originally reported in *Motyxia sequoiae* from the Xystodesmidae family, which occurs in California^14^. More recently, weak bioluminescence was also reported in other *Motyxia* species^15^. Besides bioluminescent species, the Xystodesmidae family also includes a diversity of colorful millipedes which release hydrogen cyanide and benzaldeyde from lateral glands for defensive purposes, being especially represented in Eastern United States^16,17^.

Noteworthy, bioluminescence in *Motyxia* spp is cuticular, does not involve a defined light organ, and involves a still poorly investigated photoprotein system which is dependent on ATP and Ca^2+^ or Mg^2+^^11,18,19^. The photoprotein, which was partially isolated by Shimomura, has ~ 100 kDa and it may involve a porphyrin chromophore being very unstable in solution^11,19^. Furthermore, besides the cuticular bioluminescence, *Motyxia* also displays strong cuticular blue-green fluorescence under UV light, but it is not clear whether there is or not a relationship between bioluminescence and fluorescence in these millipedes. Similar fluorescence has also been reported in the non-bioluminescent xystodesmid train millipede, *Parafrontaria laminate,* from Japan^20^. The fluorescent compounds isolated from the cuticles of *Motyxia sequoiae* and *Parafrontaria* millipedes were identified as pterin-6´carboxylic and 7,8-dihydropterin carboxylic acids^20,21^. The authors suggested that these compounds could be the putative emitters of *Motyxia* bioluminescence based on the fluorescence, and also on chemiluminescent properties in the presence of hydrogen peroxide, but no conclusive experiment has been shown yet regarding chemiluminescence of such compounds. Furthermore, the spectral properties of the bioluminescence (green) and of the fluorescence (blue-green) in *Motyxia* do not match, which would be expected if these compounds functioned as the bioluminescence emitters. Thus, whether there is a relationship between the blue fluorescence these pteridin carboxylic acids and the greenish bioluminescence in millipedes remains unclear.

**Figure 1:**
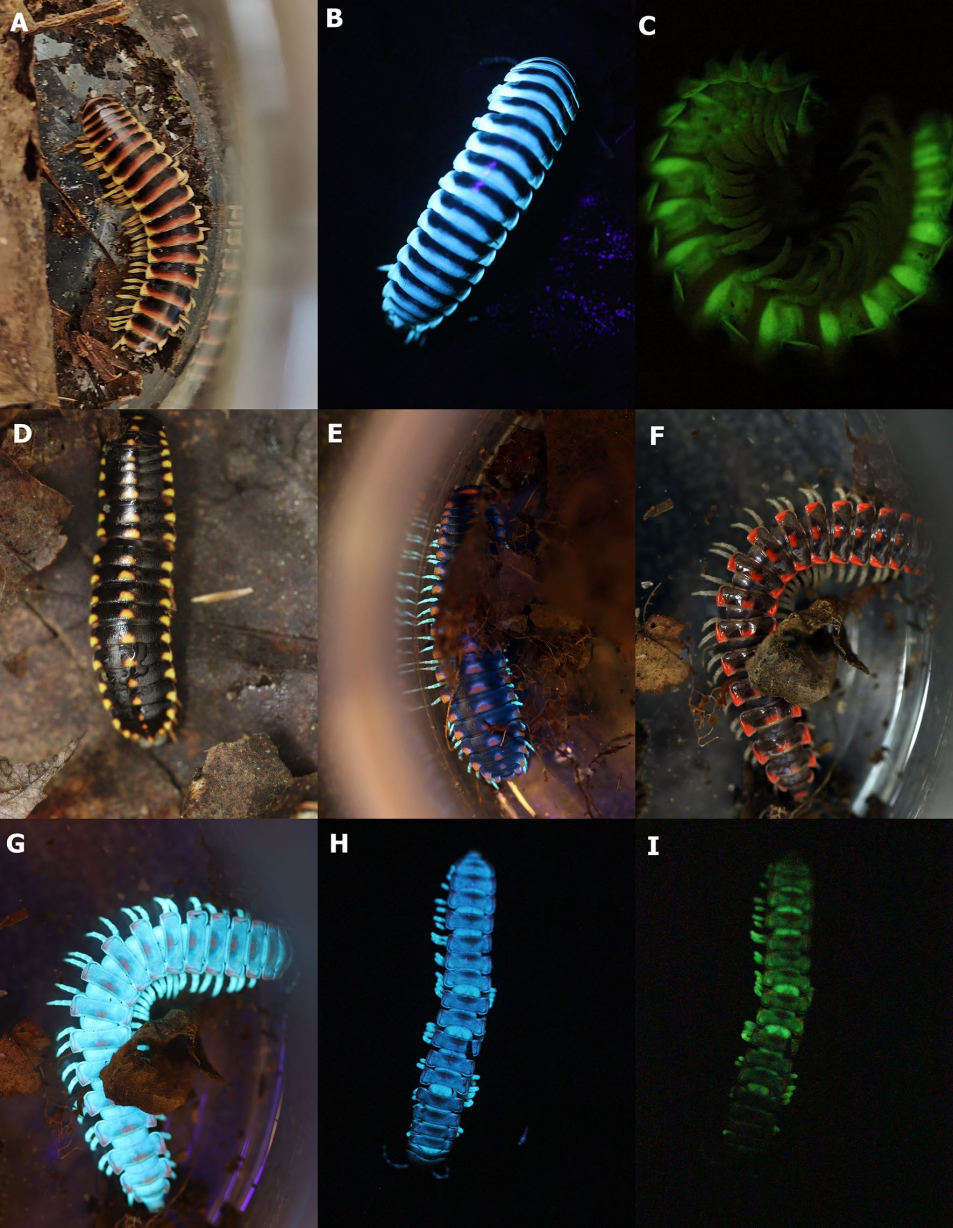
Highlands Biological station (HBS) millipedes: (**A**) *Deltatoria bremleii*; (**B**) *Deltatoria bremleii* displaying dorsal fluorescence upon UV irradiation*;* (**C**) *Deltatoria bremleii* lateral body phosphorescence*;* (**D**) *Deltatoria sp* fluorescence*;* (**E**) *Deltatoria sp* showing only legs fluorescence*;* (**F**) *Euryurus orestes;* (**G**) *Euryurus orestes* displaying blue fluorescence*;* (**H**) *Euryurus orestes* dorsal blue fluorescence*;* (**I**) *Euryurus orestes* dorsal phosphorescence.

Very recently, when walking in the woods at night at the Biological Station of Highlands (HBS) in North-Carolina with a LED UV lantern, I found 3 species of Xystodesmidae millipedes that are strongly fluorescent, emitting a blue-green fluorescence (Fig. 1). To my surprise, after turning *off* the UV light source, these millipedes also displayed a rapidly vanishing phosphorescence (Fig. 1). In this note, I report the first characterization of this phosphorescence and also of the fluorescence of these millipedes, and compare these photophysical properties with the bioluminescence properties of the xystodesmid millipede relative, *Motyxia sequoiae*, kindly provided by J.W Hastings^†^ in 2005. To our knowledge, this is the first report of occurrence of phosphorescence in animals.

## Results and discussion

### Field observations of millipede fluorescence and phosphorescence

When walking at night in the late April and beginning of May spring at HBS, 3 strongly fluorescent millipedes were readily observed upon turning an UV LED lantern. These species were identified as *Deltotaria bremleii, Deltotaria* sp and *Euryus orestes* (Fig. 1). During the day, these millipedes were found in the woods hidden under the litter and near and under decayed logs in the forest. At night they were easily found by their strong bluish fluorescence upon turning on a LED UV lantern. Upon touching them, they release from lateral glands a defensive cyanide secretion with characteristic almond fragrance. In *Deltoria* sp, only the legs and antennae fluoresced, whereas in *D. bremleii* and *Euryurus orestes* the dorsal cuticle exhibited strong fluorescence. The fluorescence of wandering millipedes at night could also be filmed with a cell phone video camera (Video-1). Noteworthy, I observed that after turning *off* the UV lantern, the millipedes still phosphoresced for less than 1 s.

### Anatomical location of fluorescence and phosphorescence

Back in the lab, I could photograph the fluorescence (Fig. 1) and phosphorescence. The fluorescence is bluish and arises from both the dorsal and ventral cuticles of the body in these millipedes. However, in the black-reddish specimens of *Deltoria bremleii* the blue fluorescence was more intense in the reddish/yellowish parts of the dorsal segments (Fig. 1A, B), and less intense in the dark parts. In of *Deltotoria sp*, only the legs and antennae displayed intense fluorescence (Fig. 1D, E). In *Euryurus orestes* the fluorescence was intense in most of the dorsal cuticle, and weaker in the reddish marks (Fig. 1G, H). The blue fluorescence could be more easily seen from the whitish areas, especially the legs and ventral tegument (Fig. 1F).

The phosphorescence of these millipedes was usually more intense in the whitish intersegmental and ventral areas, and in the legs. In *Deltatoria bremleii*, the more intense phosphorescence was usually detected in the intersegmental parts and legs, whereas in *Euryurus orestes* it was detected over all ventral area and almost all the dorsal part of the segments, with the exception of the reddish spots (Fig. 1I). Therefore, there was a good match between the anatomical location of fluorescence and phosphorescence in the dorsal part of the segments as well as the legs (Fig. 1H and I), an indication that the compounds responsible for fluorescence and phosphorescence could be the same.

### Fluorescence and phosphorescence spectra

**Figure 2:**
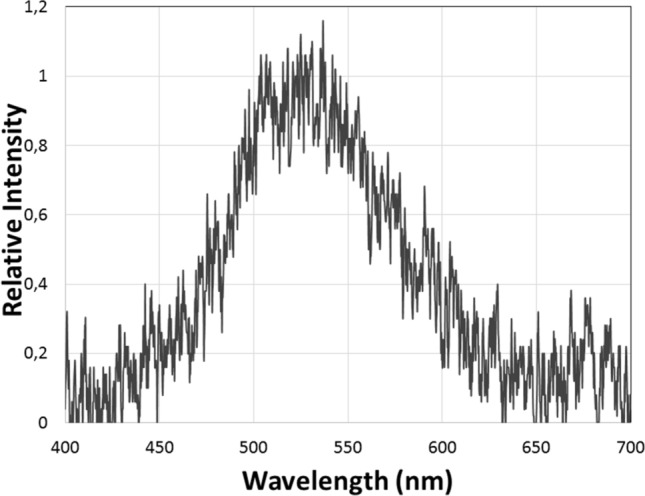
Phosphorescence spectrum of *Deltatoria bremleii* ventral cuticle and pellet of extracted material. The phosphorescence was obtained by short irradiation with UV LED lantern (370 nm), and shortly after luminescence spectra scanning.

The phosphorescence decay is very fast: it visually decays completely in less than 1 s. By using a spectrofluorometer and also a very sensitive CCD based spectroluminometer, we were able to scan the emission spectrum of the rapidly vanishing phosphorescence of the cuticle after UV light irradiation. The emission was in the green region, with a peak at 525–530 nm (Fig. 2; Table 1). Therefore, the emission spectrum of this luminescence after turning *off* the UV light source was red-shifted in relation to the fluorescence spectrum, indicating that this luminescence is indeed phosphorescence. Delayed fluorescence is unlikely considering the red-shifted spectrum in relation to fluorescence.

**Table 1:** Comparative photoluminescence and chemi- and bioluminescence spectral properties of millipedes.

Luminescence/λmax (nm)	Motyxia sequoiae*	*Deltatoria bremleii*	Euryus orestes
Phosphorescence (λ_Ph_ )			
Cuticle	–	525	520
Pellet	–	525	525
Frozen supernatant	498	–	507
Fluorescence			
λ_EX_	–	251/364	251/364
λ_FL_ (H_2_O)	467	453 (467)	459
λ_FL_ (metanol)	454	447	450
Chemiluminescence (λ_CL_ )^†^	–	513	–
Bioluminescence (λ_BL_ )^‡^	507	–	–

*We did not test *Motyxia* cuticle for phosphorescence.

^**†**^Chemiluminescence of concentrated methanol extracts in DMSO with potassium superoxide.

^**‡**^Bioluminescence of crude extracts in the presence of MgSO_4_ and ATP.

### Fluorescence and phosphorescence properties of extracted material

**Figure 3:**
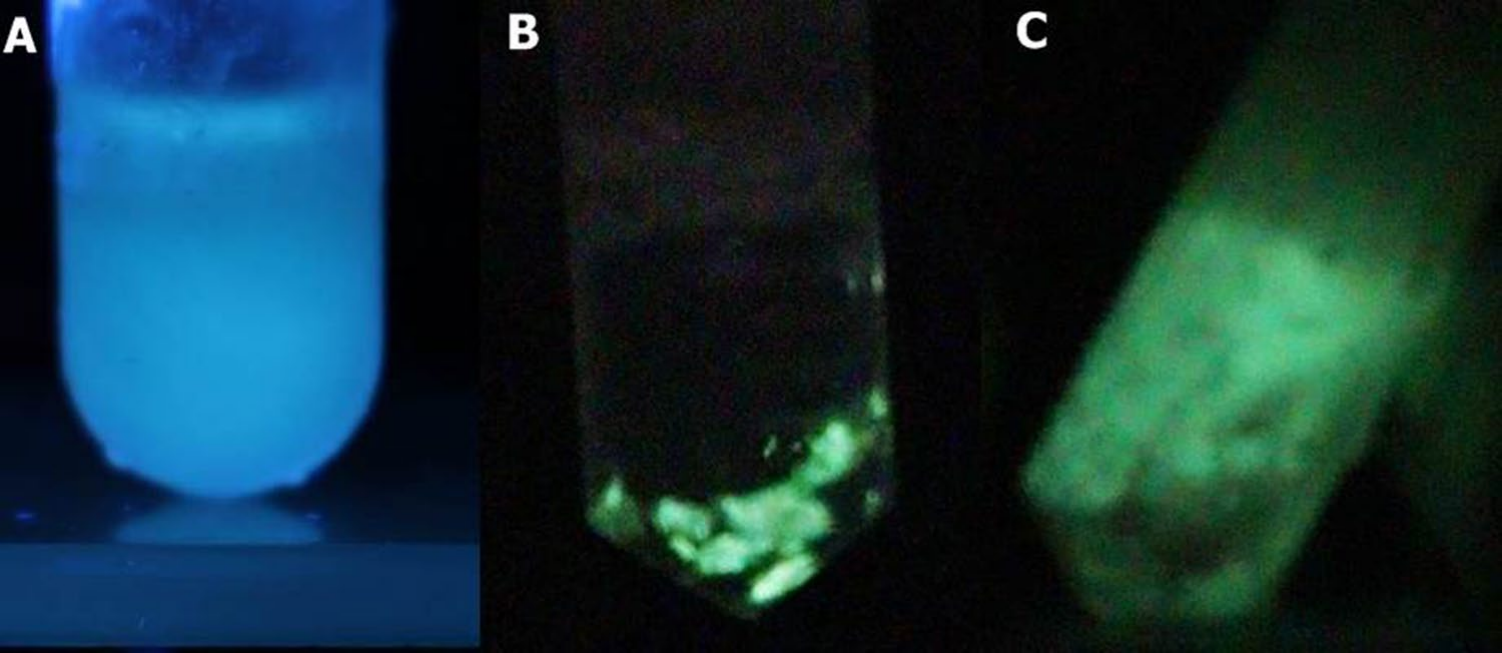
Phosphorescence and fluorescence of *Deltatoria bremleii* extracted material upon UV light irradiation: (**A**) fluorescence; (**B**) phosphorescence of pelleted material and debris after UV light irradiation; (**C**) phosphorescence of frozen supernatant.

**Figure 4:**
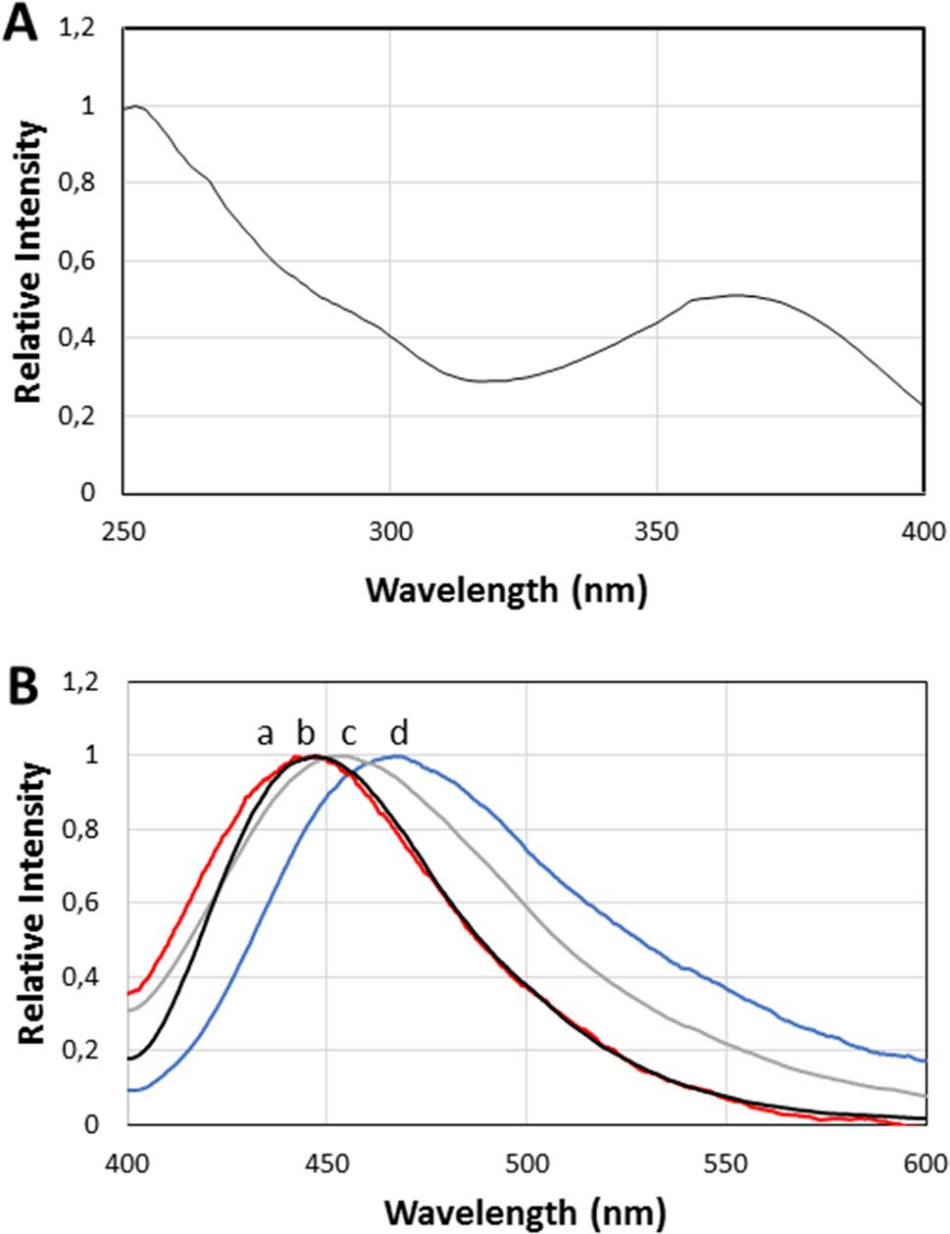
Fluorescence spectra of *Deltatoria bremleii* millipedes cuticle extracts: (**A**) excitation spectrum; (**B**) fluorescence spectra excited at 364 nm: (**a**) methanol extract of ventral cuticle; (**b**) methanol extract of dorsal cuticle; (**c**) aqueous extract of dorsal cuticle; (**d**) aqueous extract of ventral cuticle.

Upon extraction of *Deltatoria bremleii* and *Euryus orestes* cuticles and centrifugation, intense bluish fluorescence was recovered in both aqueous and acidic methanol supernatants (Fig. 3). The fluorescence spectra of aqueous extracts had excitation peaks at 251 and 364 nm, and emission at 468 nm (Fig. 4; Table 1). In methanol, although the extracted pigments displayed similar excitation spectra of the aqueous extracts, the emission spectrum was slightly blue-shifted peaking at 450 nm. The fluorescent pigments were highly soluble in water and methanol, but could not be efficiently acid extracted in ethyl acetate, indicating that such compounds are highly polar. The residue obtained upon solvent evaporation of methanol extracts was whitish for the ventral cuticle and reddish for the dorsal cuticle, and in both cases displayed strong blue fluorescence.

On the other hand, phosphorescence always remained in the pelleted material and decanted debris (Fig. 3). As expected, both aqueous and methanol extracts did not display any visually observable phosphorescence, even when oxygen was removed by slight vacuum made inside a blood collecting type vessel, using a hypodermic syringe. However, the fluorescent aqueous extracts of these millipedes, once frozen in dry ice, displayed intense phosphorescence in the green region (Table 1). Under such conditions we could also measure the life time of phosphorescence of frozen extracts, which averaged ~75 ms. Once again, these results confirm that the delayed light emission is true phosphorescence and not delayed fluorescence and the possibility that the pigment responsible for the fluorescence and phosphorescence could be the same compound, however phosphorescence is displayed only in the solid state of the cuticle or frozen crystals.

### Pigment partial isolation and properties

**Figure 5:**
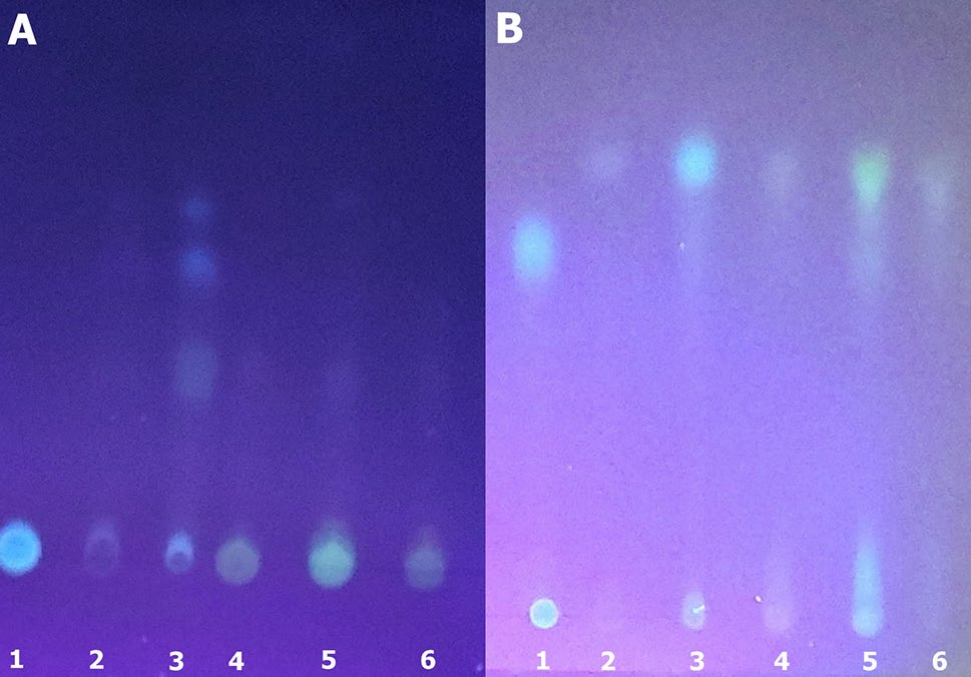
TLC of fluorescent pigments on silica-gel plates after UV light irradiation: (**A**) Ethyl acetate/Ethanol/ H2O (5:2:3); (**B**) Methanol 60%. (1) 6´-pteridinic carboxylic acid; (2) methanol/TFA extract of *Deltatoria* ventral cuticle; (3) methanol/TFA extract of *Deltatoria bremleii* dorsal cuticle; (4) aqueous extract of *Deltatoria bremleii* ventral cuticle; (5) aqueous extract of *Deltatoria bremleii* dorsal cuticle; (6) aqueous extract of *Euryus orestes* cuticle.

The methanol extracts and aqueous extracts were submitted to TLC to separate the compounds in the fluorescent spots. Under Ethyl acetate/ethanol/H_2_O as mobile phase, the *Deltatoria bremleii* dorsal cuticle extracts displayed at least 4 fluorescent spots, two with higher migration coefficients displaying blue fluorescence, and two almost merging bands with lower Rf displaying blue-green fluorescence spectra upon excitation at 364 nm (Fig. 5). The ventral cuticle displayed mainly a pigment with blue-green fluorescence with lower Rf. On the other hand, when using 60% methanol as a mobile phase, the TLC of both ventral and dorsal cuticle extracts displayed a main blue fluorescent spot with very high Rf, and a smear with the same Rf of commercial 6-pteridinic acid (Table 2). It is also noteworthy that a considerable amount of the blue fluorescent material was retained at the origin of TLC. Previously, Kuse et al. isolated from the bioluminescent millipede *Motyxia sequoiae*^20^ and from the Japanese fluorescent train millipede *Parafrontaria laminate*^21^ two main fluorescent compounds with similar fluorescence spectral properties and structures, 7,8-dihydropterin-6-carboxylic acid and pterin-6-carboxylic acid. The fluorescence properties of the eluted compounds from the blue fluorescent spots from *Deltatoria bremleii* and *Euryus orestes* millipedes, are similar to those of 7,8-dihydropterin-6-carboxylic acid and pterin-6-carboxylic acid, suggesting that these compounds could be similar to pteridine compounds.

**Table 2:** Migration (Rf) and Fluorescence properties of TLC partially isolated compounds from millipede cuticles.

Solvent System/Fluorescent spots	*Deltatoria bremleii*		*Euryus orestes*	*Motyxia sequoiae*	
	Rf	λ_FL_ (nm)§	Rf	Rf	λ_FL_ (nm)§
Solvent I*					
Spot 1	0.69	449	0.69	0.72	443
Spot 2	0.57	456	0.57		
Solvent II**					
Spot 3	0.57	449	0.57	–	–
Spot 2	0.52	456	0.52	–	–
Spot 1	0.42	465	0.42	–	–

*Solvent system I: Methanol 60%.

**Solvent system II: Ethyl acetate/Ethanol/H_2_O (5:3:2). §Peak of fluorescence spectra of water eluted compounds.

### Comparison of photoluminescence and bioluminescence in millipedes extracts

**Figure 6:**
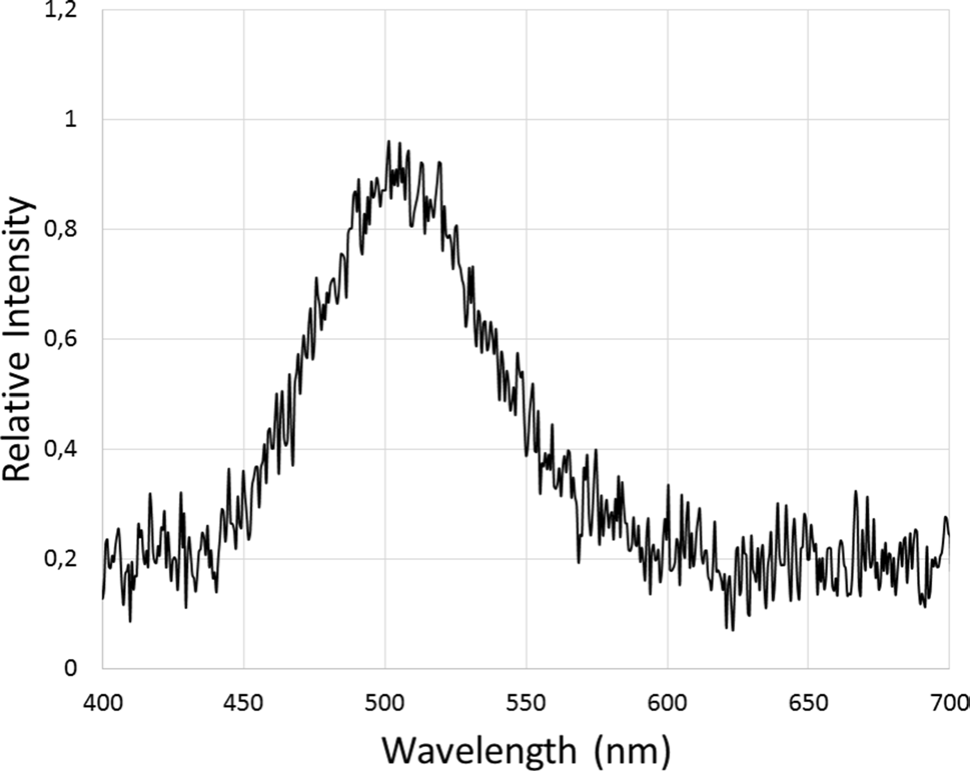
In vitro bioluminescence spectrum of *Motyxia sequoiae* crude extracts.

The fluorescence and bioluminescence of *Motyxia* have been already studied^11^. The thawed millipedes produced weak bioluminescence and also displayed fluorescence when irradiated with UV. Unfortunately, at the time we investigated the phosphorescence of the millipedes *Deltotaria* and *Euryus*, we had already run out of *Motyxia sequoiae* millipedes, which hampered the possibility to further investigate whether their cuticles were phosphorescent too. However, similarly to *Deltatoria* and *Euryus* aqueous extracts, stored frozen *Motyxia sequoiae* cuticle aqueous extracts were also phosphorescent upon UV light irradiation. As expected, the crude extracts of *Motyxia sequoiae* cuticle produced weak greenish bioluminescence in the presence of ATP and magnesium or calcium ions (Table 1). The peak of the in vitro bioluminescence spectrum of *Motyxia sequoiae* was 507 nm (Fig. 6), just in the green region. No light emission, however, could be luminometrically detected using non-bioluminescent *Deltatoria* and *Euryus* millipedes cuticle extracts under the same conditions, indicating that these millipedes do not have an active photoprotein. Furthermore, the cross-reaction between *Motyxia sequoiae* photoprotein active cold extracts and *Deltotaria* cuticle hot extracts were negative for the bioluminescence reaction.

### Chemiluminescence properties of millipedes cuticles

**Figure 7:**
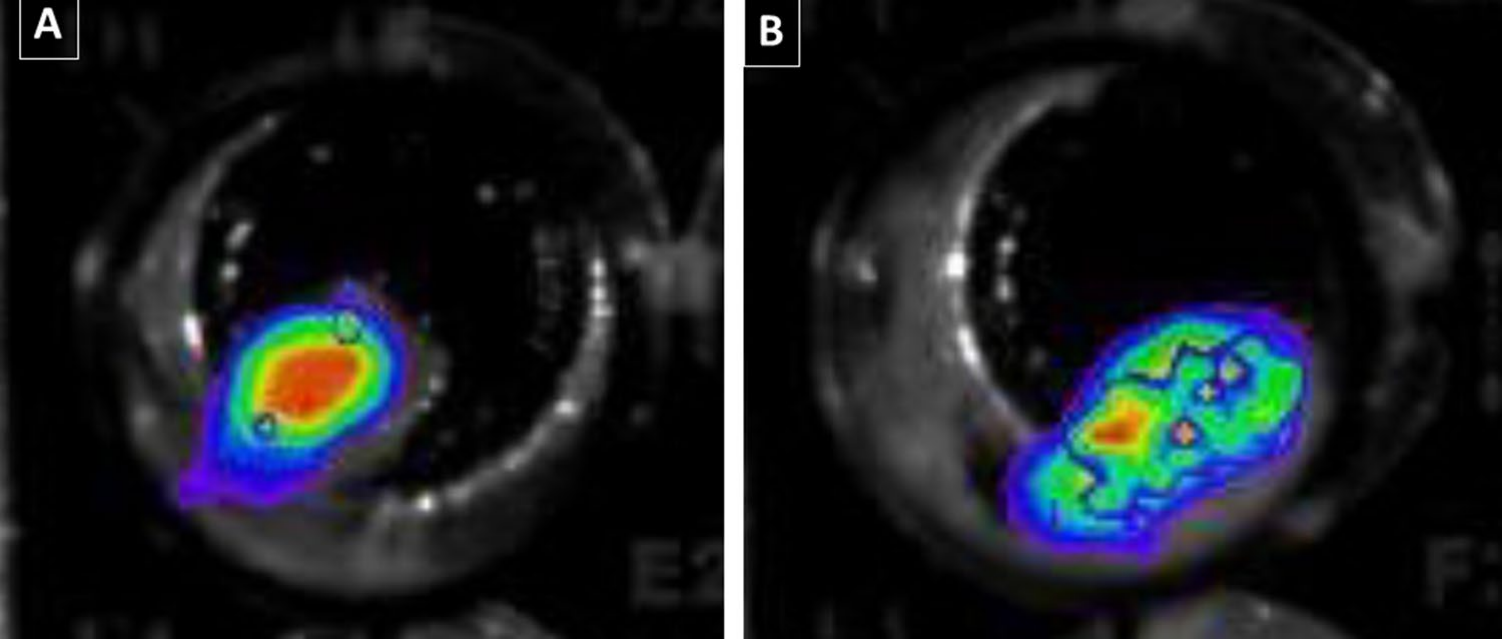
Chemiluminescence of *Deltatoria bremleii* millipedes cuticles precipitated debris: (**A**) upon addition of H_2_O_2_ and (**B**) upon addition of potassium superoxide.

Despite being non-bioluminescent, the *Deltatoria bremleii* and *Euryus orestes* millipedes cuticle extracts, similarly to the Japanese millipede *Parafrontaria laminate*^21^, produced a very weak chemiluminescence, just above the detection limit of the luminometer (Table 1), in the presence of 0.1–3% H_2_O_2_, indicating the presence of chemiluminescent compounds, similarly to the report of Kuse et al.^21^. The pelleted material also displayed chemiluminescence upon addition of 0.1–1% H_2_O_2_ or potassium superoxide, which could be detected using a highly sensitive CCD camera (Fig. 7). The concentrated methanolic extracts, once dissolved in DMSO in the presence of potassium superoxide, produced weak chemiluminescence which was intense enough to allow the measurement of the emission spectrum using a sensitive spectroluminometer, displaying an emission peak at 507 nm. Although the chemiluminescence under such harsh circumstances could not be easily attributed to a specific reaction of the isolated pigments yet, the chemiluminescence spectrum (507 nm) considerably overlaps with the phosphorescence and bioluminescence spectra of these millipedes, suggesting that this chemiluminescence may arise from the oxidation of the same compounds responsible for phosphorescence and bioluminescence. Further studies are undergoing to better understand the origin of such chemiluminescence.

### Possible biological function for fluorescence and phosphorescence in millipedes

Recently, biofluorescence has been suggested to play important signaling ecological role in crepuscular-nocturnal mammals^23^. In nocturnal millipedes, both fluorescence and phosphorescence may have not a readily recognizable biological function, considering they are blind, ruling out an inter-specific function such as sexual attraction, and because they are active just at night, when there is not strong illumination to excite the fluorescence and phosphorescence. Similarly, the coloration may not have an apparent biological function for the same reasons. However, in the case of *Motyxia* spp millipedes, it was recently demonstrated that bioluminescence has an aposematic function since that millipedes turned dark upon artificial painting their cuticles with black ink were much less prone to predation^24^. Thus a strong pigmentation could be also indicative of an aposematic defensive function, and a biological function could be hypothesized for coloration and fluorescence during the day or twilight, whenever the millipede is exposed to sunlight by a predator, showing an aposematic coloration which could be amplified by fluorescence, similarly to some bird´s plumage. Another possibility that would be worth investigating is whether there is a function during moonlit nights. Are those millipedes more active during such nights? Is moonlight, especially during a full moon, bright enough to excite this fluorescence and phosphorescence? The moonlit emission spectrum certainly has a considerable proportion of near-UV light that can potentially excite both fluorescence and phosphorescence in these millipedes. In such cases both fluorescence and phosphorescence could amplify visual warning aposematic signals that may deter a predator: after all these millipedes produce and secrete cyanide whenever disturbed, giving off their characteristic and pleasant, albeit dangerous, and sweet almond smell!

### Relationship between phosphorescence, fluorescence and bioluminescence in millipedes

Independently of whether or not there is a biological function for the observed phosphorescence in millipedes, such photophysical processes are interesting and may have a relationship with the bioluminescence mechanism of the relatively close relatives *Motyxia* spp of the Xystodesmidae family. It is noteworthy that the phosphorescent spectra of *Deltatoria* and *Euryus* cuticles, as well as *Motyxia sequoiae* frozen extracts, in the green region, considerably overlap with the bioluminescent spectrum of *Motyxia sequoiae*.

It is generally considered that all known bioluminescence examples arise from chemiluminescent reactions that yield mainly excited singlet species (fluorescent state). This is valid and expected for liquid state solvent environments, such as the cytoplasm of many photocytes where most luciferin-luciferase enzymatic reactions occur, in which the lifetime of the excited state shall be extremely short to be emissive. On the other hand, triplet excited species, with the exception of ultraweak chemiluminescence^2,22^, have not been usually considered reasonable candidates for the highly efficient bioluminescent reactions, because of their long life-times that makes them easily quenched by oxygen and other molecules in solution rather than decaying emitting light.

Similarly to the *Deltatoria* and *Euryues* phosphorescence, *Motyxia sequoiae* bioluminescence is also strictly associated with the cuticle which is an almost solid-state tissue, and not with soft tissues. In such case, triplet species may have a chance to be emissive, giving *off* phosphorescence instead of being quenched, as we have shown for the first time in the cuticles of the non-bioluminescent related species *Deltatoria* spp and *Euryus orestes* from the same subfamily. It is likely that *Motyxia sequoiae* cuticle is also phosphorescent, as indicated by their frozen extracts, although this property may have been overlooked in live millipedes.

Therefore, here I raise the possibility that, in the case of millipedes of the Xystodesmidae family, bioluminescence could be associated with the generation of triplet excited species and that the emissive process of such chemiluminescence could be phosphorescence instead of fluorescence. Alternatives could be delayed fluorescence or sensitized triplet-singlet luminescence. However, such possibilities are unlikely, considering the red-shifted spectrum of phosphorescence that argues against delayed fluorescence, and the closely matching of the bioluminescence spectrum of *Motyxia* with the phosphorescence spectrum of the related *Deltatoria bremleii* and *Euryus orestes*, that argues against an energy transfer process which requires a higher energy phosphorescence spectrum donor to sensitize the fluorescent emitter.

The photoprotein responsible for the bioluminescence reaction in *Motyxia sequoiae* has been isolated in the active form in solution, but is very unstable, indicating that it is more likely an insoluble protein which could be incorporated in the cuticle during the sclerotization process. In arthropods, the process of sclerotization and hardening of cuticles involve oxidation by reactive pigment intermediates, such as quinones and pteridines, that may react with proteins, thereby generating excited species. Although the active photoprotein has been partially isolated in the soluble fraction, which in principle argues against the participation of emissive triplet excited states, the definition of photoprotein itself does not exclude such possibility. The photoprotein is a protein that oxidizes the substrate, stabilizing a peroxide intermediate, which in the presence of triggering agents such as calcium, undergoes conformational changes promoting its breakdown and generating an excited product that decays emitting light just once, without apparent turnover like a typical luciferase^1^. Furthermore, the relatively efficient enzyme generation of triplet species chemiluminescence is not an unlikely possibility, it has been already demonstrated in vitro for the HRP/Isobutanal system which produces triplet acetone by Cilento and coworkers^22^. In such a circumstance, a triplet excited product generated inside a closed, relatively rigid and protective environment against solvent quenching of a protein associated to the cuticle could be in principle emissive. Studies are awaited to investigate this interesting possibility.

## Concluding remarks

Here I report for the first time the occurrence of phosphorescence in the cuticles of fluorescent millipedes, *Deltoria* spp and *Euryus orestes* occurring in North Carolina forests, being also the first report characterizing phosphorescence in arthropods and animals. The phosphorescence of these millipedes arises from the cuticle, has a short life time and its emission spectrum in the green region is red-shifted as compared with the spectrum of its fluorescence. This phosphorescence may arise from pteridine acid derivatives in the cuticle that may also be responsible for the fluorescence. Remarkably, the considerable overlapping of the phosphorescence spectrum of these millipedes cuticles and frozen extracts with the bioluminescence spectrum of the close relative *Motyxia sequoiae* and its extracts raises the possibility, for the first time, that bioluminescence in millipedes could be generated by a chemiluminescent reaction generating preferentially triplet excited states (phosphorescent state) instead of singlet states (fluorescent state).

## Material and methods

### Millipedes

Frozen *Motyxia sequoiae* millipedes, previously obtained from California, were graciously provided by J. W. Hastings^†^ at Harvard University in 2005 and used in our laboratory for biochemical studies. Fluorescent millipedes *Deltatoria brimleii, Deltotaria sp* and *Euryus orestes* were collected during May of 2019 and 2023 at the Highland Biological Station (Highlands, NC) and were kindly identified by Derek Merret (Virginia Technology Natural History Museum).

### Photography

Photographic images and videos of millipede fluorescence were obtained using a Galaxy S10Plus camera. Images of millipede phosphorescence were obtained using a Canon Ti5 camera with a 100 mm macro lens, and 10 s exposure at 12,200 ISO after UV irradiation using a SouthWalker lantern provided with Nichia UV LED (Japan) during 10 s.

### Fluorescence and phosphorescence spectra

Fluorescence and phosphorescence spectra were obtained using mainly a Hitachi F4500 spectrofluorometer (Japan). Fluorescence spectra were obtained upon excitation at 364 nm with an excitation window of 2.5–5.0 nm and an emission window of 10 nm. Phosphorescence spectra were scanned from 400 to 700 nm with excitation at 364 nm, excitation window at 10 nm and speed 240 nm/min, using the phosphorescence mode. Bioluminescence and phosphorescence spectra were also obtained with a refrigerated CCD camera provided spectroluminometer LumiSpectra (ATTO, Japan). Phosphorescence spectra in this latter equipment were measured in the high sensitivity mode upon pre-irradiation with a SouthWalker lantern provided with Nichia UV LED (Japan) followed by turning off the UV light source.

### Phosphorescence life-time

The phosphorescence life-time was measured using a Hitachi F4500 spectrofluorometer in the module Time scan (Phosphorescence), upon excitation at 364 nm and emission at 525 nm.

### Cuticle extraction

#### Fluorescent compound extraction

To obtain fluorescent pigments, frozen *Deltatoria* millipides were allowed to partially thaw, and then laterally opened with a scissor. After removing the still frozen digestive tube, the remaining dorsal (pigmented) and ventral (whitish) cuticles, including legs, were separately ground with a mortar on ice using methanol/1% TFA. The suspension was then centrifuged at 15,000 g during 15 min at 4 °C, and the supernatant used to measure fluorescence spectra and for Thin Layer Chromatography (TLC) analysis. The cuticles were also ground in 50 mM sodium acetate buffer pH 5.8 supplemented with 10 mM EGTA and 0.20 M NaCl, centrifuged and the supernatants used for fluorescence and phosphorescence analysis.

#### Bioluminescence system extraction

In the case of photoprotein extraction from either *Motyxia* or other fluorescent millipedes, we followed the protocol of Shimomura^11^. The cuticles of 1 or 2 of these frozen millipedes were also excised with scissors using the procedure described above, and grounded in an ice-cold mortar, with an ice-cold extraction buffer consisting of 10 mM sodium acetate buffer pH 5.8, containing 0.2 M NaCl, 10 mM EDTA supplemented with an antiprotease cocktail (Merck). The homogenates were then centrifuged at 15,000 g for 15 min at 4 °C, and the supernatants used for photoprotein in vitro bioluminescence and chemiluminescence luminometric assays and spectra.

#### Bioluminescence and chemiluminescence assays

The in vitro bioluminescence was assayed using an adaptation of the protocol described by Shimomura^11^. Inside a luminometer tube, 10–20 µl of crude extract were mixed with a solution consisting of 80–90 µl of 0.10 M Tris–HCL buffer pH 8.5, 2 mM ATP and 4 mM MgSO4. The luminescence output was measured in counts per second (cps) using an ATTO AB220 luminometer (Tokyo, Japan). Chemiluminescence assays were performed by mixing crude and methanolic millipede cuticle extracts with hydrogen peroxide 0.1–3% in buffer and light intensities were measured using an ATTO luminometer.

#### Bioluminescence and Chemiluminescence spectra

Bioluminescence and chemiluminescence spectra were measured using a LumiSpectra spectroluminometer (ATTO, Japan). For bioluminescence spectra 10–20 µl of Millipede crude extracts were mixed with a solution consisting of 80–90 µl of 0.10 M Tris–HCL buffer pH 8.5, 2 mM ATP and 4 mM MgSO_4_ inside a luminometer tube. Chemiluminescence spectra were measured upon mixing 2–10 µl of concentrated methanolic cuticle extracts with 100 µl of a solution of potassium superoxide (8 mg/mL) dissolved in anhydrous DMSO (SIGMA-Aldrich).

#### Thin Layer Chromatography (TLC)

Thin Layer Chromatography (TLC) was performed using pre-coated silica-gel 60 sheets (Alugram XtraSIL G, Germany) using methanol 60% in water or ethyl acetate/ethanol/water (5:3:2) as mobile phases at room temperature. Fluorescence was revealed using UV irradiation at 370 nm.

## Supplemental information

Link for Video-1. Fluorescent *Deltatoria bremleii* millipede: https://drive.google.com/file/d/1TT_Pajh6MGCc8a9Q6csaWjDWFHAq-Ok_/view?usp=drive_link

### Supplementary Information

Supplementary Video 1.

Supplementary Information 1.

## Data Availability

The datasets used and/or analysed during the current study are available from the corresponding author on reasonable request.
