# Supplementary material for: Biophosphorescence in fluorescent millipedes (Diplopoda: Xystodesmidae) and its relationships with bioluminescence
Source: Sci Rep. 2023 Dec 13;13:22171. doi: 10.1038/s41598-023-47860-9 (PMC10719341; doi:10.1038/s41598-023-47860-9)
Supplement: Supplementary file 2 — Supplementary Information 1. [file 41598_2023_47860_MOESM2_ESM.docx]

**Supplementary Video Legend**

The video shows the fluorescence of *Deltotaria brimleii* millipede walking at night, upon irradiation with a portable UV lantern.
